# Theoretical Model and Actual Characteristics of Air Pollution Affecting Health Cost: A Review

**DOI:** 10.3390/ijerph19063532

**Published:** 2022-03-16

**Authors:** Xiaocang Xu, Haoran Yang, Chang Li

**Affiliations:** 1 School of Economics and Management, Huzhou University, Huzhou 313000, China; 03122@zjhu.edu.cn; 2 Research Center for Economy of Upper Reaches of the Yangtse River, Chongqing Technology and Business University, Chongqing 400067, China; 2020651005@email.ctbu.edu.cn; 3 School of Economics and Management, Hebei University of Science and Technology, Shijiazhuang 050018, China

**Keywords:** air pollution, health cost, healthy production function, bivariate model, exposure-response function

## Abstract

Background: The impact of environmental pollution (such as air pollution) on health costs has received a great deal of global attention in the last 20 years. Methods: This review aims to summarize the theoretical analysis model of air pollution affecting health costs, and further explore the actual characteristics of the impact of air pollution on health costs. The following main databases were taken into account: Web of Science Core Collection, Medline, SCOPUS, PubMed, and CNKI (China). As of 30 March 2021, we retrieved a total of 445 papers and ended up with 52 articles. Results: This review mainly expounds clarification of the concept of air pollution and health costs, the theoretical model and the actual characteristics of air pollution affecting health costs. In addition, it also discusses other related factors affecting health costs. Conclusion: Our conclusion is that, while academic research on the relationship between air pollution and health costs has made some progress, there are still some shortcomings, such as insufficient consideration of individual avoidance behavior and rural–urban and international mobility. Therefore, the simple use of the original data obtained in the statistical yearbook of the health cost caused by air pollution is also the reason for the errors in the empirical results. In addition, the choice of proxy variables of environmental pollution by scholars is relatively simple, mainly focusing on air pollutants, while the impact of water quality or soil pollution safety on health costs is becoming increasingly prominent, and will become the focus of future research.

## 1. Introduction

The causes of health problems and their costs are of urgent concern to scholars and governments around the world. Environmental pollution, such as air pollution, is the most important factor to be discussed. Air pollution is a deep cause of soaring health costs. The harm caused by air pollution to the health of residents has been proved, such as in the Great Smog in London in 1952, which shocked the world by killing tens of thousands of people. A large amount of energy consumption and the development of urbanization under the invention of the steam engine by Watt in the UK and the booming industrial revolution made air pollution more and more serious, and caused damage to human health. Therefore, a large amount of literature on the relationship between air pollution and health appeared, such as the effect of air pollutant concentration on the mortality and morbidity of different populations (Currie et al., 2009) [1]. Furthermore, the topic of research extends from the impact of air pollution on the health of residents to its impact on the increasing high health cost. Murthy and Okunade (2000) [2], Clancy et al. (2002) [3] and Brunekreef and Holgate (2002) [4] verified the positive relationship between health costs and pollutant emissions. Frogner (2008) [5], Cao and Han (2015) [6] pointed out that the growth rate of health costs increased year by year and even exceeded the growth rate of GDP. This trend has aroused the continuous attention of many scholars on the determinants of health costs. Many factors affect health costs, but the most critical factor is environmental quality (An and Heshmati 2019 [7]). Therefore, quantifying the health costs of air pollution is of great significance to the quality and efficiency of environmental and health policymaking (Chen and Chen 2020 [8]).

In brief, the international research on the impact of air pollution on health costs in the field of economics started a long time ago, but research in China has only just begun in the last decade, which lacks the deep theoretical basis and application of an empirical model with detailed discussion. Therefore, there is an urgent need for a systematic review of current relevant research to promote the development of high quality research in the future. This review aims to summarize the theoretical analysis model of air pollution affecting health costs, and further explore the performance characteristics of the impact of air pollution on health costs.

## 2. Materials and Methods

### 2.1. Search Process

The following main databases were taken into account: Web of Science Core Collection, Medline, SCOPUS, PubMed, and CNKI (China). The following combinations of terms were practiced with the phrase “and/or” to maximize the scope and type of material achieved in the search: ‘Air Pollution’, ‘Air-Quality’, ‘Carbon Emissions’, ‘Health Cost’, ‘Health Expenditure’, ‘Healthcare expenditure’, and so forth.

### 2.2. Inclusion-Exclusion Criteria and Data Extraction Process

We first used the above words or concepts to search for topics and defined the time of the literature search from 1991 to 30 March 2021. For example, 282 results for Health Expenditure (topics) and Air Pollution (topics) were found from the Web of Science Core Collection since 1991. All the above searched words or concepts combinations were found in thousands of articles. First, we eliminated the conference abstracts, editing materials, newsletters, and conference proceedings, leaving 445 articles. Second, the remaining 445 articles were simply screened for form and quality by a researcher, and 253 articles were left. Third, the author carried out in-depth quality screening for the remaining 253 articles. The screening criteria included too-short words, too-simple content, and high similarity, and so forth.

Information extracted from all included papers included: author, publication date, sample country, study methodology, study purpose, and key findings. The relevant information of the paper was exported to the Excel database through Endnote, and the duplicates were deleted. The results were initially extracted by one researcher and were then cross-checked by another researcher to ensure that all data had been filtered and reviewed. If two researchers had different opinions, the two researchers would review together until a final agreement was reached.

### 2.3. Quality Assessment of the Literature

The JBI Critical Appraisal Checklist was used to evaluate the quality of each article. The JBI (Joanna Briggs Institute) key assessment tool was developed by the JBI Scientific Committee after extensive peer review and was designed for system review. All features of the study that met the following eight criteria were included in the final summary: (1) clear purpose; (2) complete information of sample variables; (3) data basis; (4) the validity of data sorting; (5) ethical norms; (6) effective results; (7) application of appropriate quantitative methods and stating the results clearly. Method quality was evaluated by the Yes/No questions listed in the JBI Key Assessment List. On average, each article on analysis received 7 out of 8.

## 3. Results

### 3.1. Conceptual Interpretation of Air Pollution and Health Cost

The work of Narayan and Narayan (2008) [9] laid a foundation for the study and the expansion of the relationship between air pollution and health costs. After that, more in-depth and detailed studies began to emerge gradually over a decade. In the specific research, the representative variables of air pollution and health costs are not the same.

#### 3.1.1. Air Pollution

According to the International Organization for Standardization (ISO) definition, air pollution refers to the phenomenon of substances exceeding environmental quality standards and reaching a certain concentration in the atmosphere and endangering human health and the environment. Scholars have different choices of variable indexes corresponding to air pollutants, so the concept and category of air pollution should be explained. Due to the different actual conditions in different countries, even if the same measurement technology is used at the same place, the gas types and concentrations detected at different times are also different.

In view of the availability of data and the diversity of research perspectives, scholars have selected air pollution indicators, including concentration or emission air pollutants, as well as air pollutant indicators not limited to the narrow sense.

After sorting out, as shown in Table 1, the main air pollution indicators in the existing studies mainly fall into the following three categories: (1) Waste gas material index—mainly refers to the source of air pollution, such as industrial waste gas emissions: CO_2_, SO_2_, smoke dust, nitrogen oxides, inhalable particulate matter, and so forth. The emission of industrial waste gas and human respiratory diseases will also cause physiological dysfunction, mainly in industrial combustion and production processes; (2) Air quality indicators—mainly refers to the environmental impact caused by exhaust emissions, such as PM10, PM2.5, and AQI. The AQI index combines several kinds of pollutants, which can reflect the degree of air pollution more comprehensively than a single pollutant; (3) Indirect indicators—mainly refers to changes in people’s behavior or policies caused by air pollution, such as changes in the energy mix, car ownership, and the purchase of masks. The increase of private car ownership is one of the main sources of outdoor air pollution, so car ownership can be used as a proxy variable for air pollution. The volume of mask purchases reflects the cost of the avoidance actions people take to deal with air pollution. An unreasonable energy structure will aggravate air pollution.

#### 3.1.2. Health Cost

Accurate estimation of the health costs caused by air pollution is of great significance for the analysis of social costs. Due to the different research emphases, scholars at home and abroad have different choices of health cost calculation methods.

1. Adopt expenditure on medical care for urban residents. Some scholars believe that urban residents suffer more from environmental damage (such as exhaust pollution and work pressure) than rural residents, thus bearing greater health costs. Therefore, the medical care expenditure of urban residents is chosen as the research object (Li and He, 2019) [10].
2. Per capita medical visits and per capita health expenditure were adopted. Based on the traditional Chinese concept of “no treatment for minor diseases and little treatment for major diseases”, the number of visits per capita and health expenditure per capita were selected as proxy variables of health costs (Alimi et al., 2019) [11].
3. Adopt the cost of disease method. Non-empirical methods were used to calculate health cost, that is, direct medical consumption expenditures for diseases and income losses caused by inability to work due to air pollution were calculated based on hospital data (Cao and Han, 2015) [6].

In addition to the above three estimates of health costs, the researchers also estimated the total expenditure of patients with environmental diseases (An and Heshmati, 2019) [7].

### 3.2. Theoretical Model of Air Pollution Affecting Health Cost

Regarding the selection of research models for estimating the health costs caused by air pollution, there are two main categories: empirical models and non-empirical models, which are also called statistical model methods and analytical methods by some scholars (Qu and Yan, 2015) [12]. Scholars in the field of economics mainly use the empirical model to study the relationship between air pollutants and health costs by selecting sample data within a certain time range and using econometric models to explore whether the conclusions are significant or not. Here, we choose two models widely used by scholars for illustration purposes. Non-empirical models are mainly used in biomedical and public health fields to calculate the actual number of people affected by air pollutants based on the explosion–response function, which is multiplied by the economic loss per unit case to obtain the total health cost. The model is designed to illustrate the relationships between the key variables under study to better understand the real world. As economics is a social science, research problems cannot be separated from certain assumptions, and a model cannot cover all assumptions, so the conclusions explored by models are often flawed.

#### 3.2.1. Health Cost Measurement Model

The health costs of air pollution are of great interest to economists and non-economic scholars alike, and researchers have quantified the health costs of individuals exposed to air pollution.

(1) Exposure–response function

The incidence of disease and death caused by air pollution is a small probability event per unit of time. EHC210 (WHO Environmental Health Criteria 210, 1980) first established the exposure–response function as the basis of the risk assessment paradigm. Subsequently, the Poisson regression model of statistics was used to estimate the actual number of people affected by air pollution. The exposure–response function has three main forms: exponential form, linear form, and log-linear form (Zhang et al., 2007) [13]. The researchers chose the optimal form for the study based on the specific adverse health effects of air pollution in the study area. The health effects of people exposed to air pollution are expressed as follows in Equations (1) and (2):

(1) $$E=\exp\left[\beta \cdot \left(C-C_{0}\right)\right]\cdot E_{0\;}$$

(2) $$\Delta E=E_{0}\beta \left(E-E_{0}\right)\;,\;$$

where β is the coefficient of the exposure–response relationship; C and $C_{0}$ represent the environment and threshold pollutant concentration respectively; E and $E_{0}$ represent the health effects corresponding to C and $C_{0}$ respectively, often represented by the number of morbidity or deaths. ΔE is the difference of health effects under the environment and threshold concentration. In Equation (1), β, E, C and $C_{0}$ can be calculated by referring to the values of β and $C_{0}$ selected from existing studies and the current statistical data, so as to obtain the specific values of $E_{0}$, which can be further calculated by Equation (2).

The exposure–response function is somewhat controversial.

Firstly, although illness or death occurring in unit time is a random small probability event, still, the exposed-reaction function selection of samples is usually a random sampling. Still, based on a fixed population for the analysis, the conclusion is not in conformity with economic statistics random sampling principles or experimental research on its conclusion because of the cautious attitude. For example, Graff and Neidell (2013) [14] proposed that residents in severely polluted areas are not randomly assigned because individuals choose their houses based on certain preferences. In addition, due to the existence of heterogeneity among individuals, people of different ages have different perceptions of poor air quality and body functional responses. In addition, according to the research of Schwartz (2004) [15], children’s bodies are not fully developed and they are more vulnerable to physical damage when they respond to environmental shocks.

Secondly, due to the application of the exposure–response relationship and the selection of health endpoints based on hospital data, such as morbidity rate, hospitalization rate, mortality rate, and so forth, the impact of air pollution on residents is limited to physical damage. On the one hand, air pollution leads to the reduction of non-working hours such as illness and hospitalization, while the reduction of working hours reduces the income of residents. On the other hand, when air pollution is severe, residents choose to be forced to stay indoors. Taking into account the indirect effects of the above air pollution on the human body, it may cause stress and anxiety to the residents and damage their mental health. Pedersen et al. (2004) [16] confirmed that the incidence of schizophrenia is related to traffic-induced air pollution by using air pollution data of 7455 children in Denmark at birth, especially the high correlation between benzene and CO in traffic gas emissions.

Finally, Graff and Neidell (2013) [14] proposed that a major endogenous problem of the exposure–response relationship is individual avoidance behavior. The estimation of individual avoidance behavior can be divided into market perspectives and non-market perspectives. Individual defensive expenditures and willingness to improve air quality can be observed through the market, while other non-market behaviors, such as expenditures for alternative actions to avoid outdoor air pollution and other opportunity costs, are difficult to observe and estimate.

(2) Health cost estimation methods

There are various methods for calculating economic loss per unit. Kuan et al. (2005) [17] introduced in detail the commonly used methods including disease cost method, human capital method, willingness to pay method, and benefit conversion method. Although the estimation methods are different, their core is to calculate the total health costs caused by air pollution through Equation (3).

(3) $$THE=\sum (\Delta E_{i}\cdot V_{i}).$$

THE represents Total Health Expenditure, $V_{i}$ represents the economic loss per unit of disease, and i represents the different health endpoints. The endpoints of health effects were generally selected as total mortality, chronic bronchitis, respiratory diseases, and non-accidental death. This method has been applied by some scholars (Chen et al., 2010 [18]; Chen et al., 2020 [19]).

#### 3.2.2. The Empirical Analysis Model of the Impact of Air Pollution on Health Cost

Tracing back to the source of relevant studies, it is found that scholars’ studies are mainly based on the health production function initiated by Grossman (1972) [20] and the health care expenditure function (also known as the bivariate model) proposed by Newhouse (1977) [21].

(1) Health Production Function

Based on the analysis of health demand and medical care demand, Grossman (1972) [20] regarded health as capital and used the marginal analysis method to explore how health investment and deterioration rate, such as age, salary, and education level, affect health in addition to individual health endowment. Under this analytical framework, health is regarded as a depreciable capital good, and its stock changes dynamically. For example, the occurrence of disease and the increase of age will reduce the stock of health. Some subsequent studies took air pollutants as independent variables into the equation. For example, Cropper (1981) [22] regarded a single SO_2_ concentration variable as a proxy variable of air pollution and studied individual willingness to pay for air quality improvement. Gerking and Stanley (1986) [23] studied the relationship between health and air pollution exposure in 824 adult workers in St. Louis. After adding the air pollution variable, the health demand function can be expressed as:

(4) $$\ln HD_{t}=a_{1}\ln C_{1}+a_{2}\ln W_{t}-a_{3}t-a_{4}\ln P_{t}-a_{5}\ln G_{t}-a_{6}\ln PM+\;\delta_{1}lnKh_{1}t+\ldots +\delta_{n}Kh_{n}t+\varphi_{1}$$

Equation (4) represents the health demand level $HD_{t}$ when age is t. Where $C_{1}$ is a constant, $W_{t}$ is the wage rate, $P_{t}$ is the concentration of air pollution in the environment where consumers live, $G_{t}$ is the personal lifestyle, Pm is the price of medical services, $Kh_{n}t$ is the educational variable. Together, these factors determine an individual level of health needs.

However, the Grossman function and its extensions study the variables determining health capital and how they affect health. As individual differences and group characteristics make health measurement more complex, Miao and Chen (2010) [24] pointed out that scholars gradually take health cost expenditure as the alternative observation variable of health level to explore the relationship with air pollution. However, there are still relatively few studies in this field. Yang et al. (2013) [25] studied the urban population data of 29 provinces and regions from 1998 to 2010 by using the VAR model of panel data with interactive effects as the alternative variable of health cost expenditure and concluded that the cost of environmental pollution in each region accounted for about 8%–10% of the per capita real GDP. Qi et al. (2015) [26] used this alternative method for reference and used the spatial lag model to study and concluded that environmental capacity had a significant impact on the health expenditure of residents in central and western China, and the regression coefficient was negative. It indicates that the stronger the capacity of environmental capacity to dissolve pollutants, thus reducing the health costs of residents.

The health production function (Grossman, 1972) [20] is widely used, but it also has some shortcomings. First of all, when scholars apply the health production function, several indicators, such as mortality rate or the mortality rate of children under five years old, are selected to measure the health level (Arthur et al., 2017 [27]; Sun and Li, 2017 [28]). However, with the progress of medical technology and the improvement of health awareness, such as regular physical examinations every year and timely medical treatment, the study focusing only on mortality cannot reasonably measure the real health level of residents. Cutler et al. (2006) [29] mentioned that the number of hospitalizations caused by environmental pollution increased, but the absolute mortality rate changed less, because the survival rate increased with the development of economic society and the progress of medical technology. Second, there is a gap between the health production function and people’s actual living conditions when it is applied. Separating from the analysis of health production functions by economists, health capital is not discrete for individuals. Therefore, Zhao (2005) [30] pointed out that the description of one unit of health is unrealistic, and the study of individual demand for health services is more in line with the public demand for health.

(2) Bivariate Model

Income can explain most of the differences in per capita health costs based on the sample data of 16 different countries (Hraim, 1974) [31], Newhouse (1977) [21] proposed a bivariate model: Through linear regression of per capita GDP and per capita healthcare expenditure, the determination coefficient R^2^ = 90 indicates that the variation of health costs can be explained by per capita income as high as 90%. This classic conclusion has attracted the attention of scholars, and the existence of a certain relationship between income and health costs has become one of the accepted viewpoints of health economics (Parkin et al., 1987) [32]. Gerdtham and Jonsson (1991) [33] expressed the bivariate model of per capita health care expenditure (HEPC) and per capita GDP as Equation (5):

(5) ln(HEPC)=α+βln(GDP)+δ. 

However, as the main proxy variable of environmental pollution, the air pollution variable does not enter into the equation affecting the health costs at the beginning but has gone through a certain stage of development. Matteo and Matteo (1998) [34] and Karatzas et al. (2000) [35] extended the bivariate model to the multivariate model, taking into account other social cost factors of health costs: age, level of medical services, number of practicing doctors, income distribution, proportion of population over 65 years old, female labor force participation rate, and so forth. With economic and social development, scholars found that the effect of air pollution on health costs—for example, Jerrett et al. (2003) [36] used a continuous two-stage regression model, cost analysis in Ontario, Canada—may affect the possibility of a relationship between variables, and endogenous, toxic pollution output and total health costs were positively correlated. For the first time, Narayan and Narayan (2008) [9] added environmental factors to the bivariate model to explore the role of environmental quality in health costs in the short and long run in eight OECD countries. The multivariate model established by the authors is as in Equation (6):

(6) $$\ln HE_{t}=\alpha_{0}+\alpha_{1}\ln Y_{t}+\alpha_{2}\ln NI_{t}+\alpha_{3}\ln SU_{t}+\alpha_{4}\ln CA_{t}+\varepsilon_{t}$$

where HE is per capita health expenditure, Y is real per capita income, NI is nitrogen oxide emissions, SU is sulfur oxide emissions, CA is carbon monoxide emissions, and $\varepsilon_{t}$ is the random error term. Using the panel co-integration approach, the authors find overwhelming evidence that there is panel co-integration between per capita health expenditure and its determinants.

Bivariate and its extended model have developed rapidly in the last ten years. The sources of air pollution with high health costs can be looked at in different ways. For example, the spatial Dupin model is analyzed from the perspective of a time lag effect and a space spillover effect (Li and He, 2019) [10]; Bayesian quantile regression (BQR) can study the conditional-response distribution in regression (Xu et al., 2019) [37]; and the fixed-effect model can produce unbiased and consistent estimators (Cui et al., 2016 [38]; An and Heshmati, 2019 [7]). Table 2 summarizes a partial review of studies on the relationship between air pollution and health costs based on bivariate and its extended model.

### 3.3. Actual Characteristics of Air Pollution Affecting Health Cost

Reviewing the research results of global scholars, the characteristics of the impact of air pollution on health costs can be summarized as the following three points.

#### 3.3.1. Significant Co-Correlation

Air pollutants in the atmosphere can lead to increased health costs for residents. Researchers have reached similar conclusions based on data from different countries using different econometric models. Hao et al. (2018) [49] used the GMM estimation method, Ouyang H et al. (2017) [43] used the spatial panel econometric model, Ceylan (2020) [42] used the Bayesian optimization support vector regression model, and so forth. It is concluded that the deterioration of air quality will stimulate residents’ health costs by affecting their health level. For example, Alimi et al. (2019) [11] estimated the impact of per capita carbon emissions on health expenditure in ECOWAS from 1995 to 2014 using three methods and found that at the level of 5% of conventional health costs, the positive value of the carbon emission coefficient was significant, indicating that CO_2_ emissions increased the health costs of ECOWAS residents.

#### 3.3.2. Time Lag

The change of health costs has a time lag. Specifically, air pollution has different long-term and short-term impacts on health cost. Raeissi (2018) [50] found through data from Iran from 1972 to 2014 that from a long-term perspective, air pollutants have a positive and significant impact on health costs (for every 1.00% increase in carbon dioxide index, public health expenditure and private health expenditure will increase by 3.32% and 1.16% respectively), while from a short-term perspective, the relationship between the average concentration of air pollutants and health costs is not significant, but has a certain time lag effect. Yahaya et al. (2016) [41] and Li and He (2019) [10] found, based on the data of 125 developing countries from 1995 to 2012 and China from 2002 to 2015, that the impact of PM2.5 air pollution on per capita health expenditure increases with time, and the time lag effect is significant.

#### 3.3.3. Category Difference

Different air pollutants have different effects on health cost. Apergis et al. (2018) [51] found that carbon dioxide (CO_2_) emissions have the relatively strongest impact on health care, particularly in countries with high health care spending. Miao and Chen (2010) [24] believe that PM10 has a greater effect on individual health costs in China, but Zhao (2020) [52] finds that, compared with PM2.5 and PM10, O_3_ pollution has a greater effect on personal medical spending, and every 10% increase in O_3_ pollution concentration will increase individual health costs by 14.6%. Furthermore, Narayan and Narayan (2008) [9] examined the role of environmental quality in determining per capita health expenditure using data from eight OECD countries from 1980 to 1999, respectively, in the long and short term. In the short term, CO emissions had a statistically significant positive effect on health expenditure. In the long run, SO_2_ emissions also have a statistically significant positive impact on health spending.

### 3.4. The Role of Other Factors in the Impact of Air Pollution on Health Cost

The relationship between air pollution and health costs is not a simple binary relationship. In the complicated reality, it is necessary to study the quantitative relationship between air pollution and health costs from different perspectives to make the research more reasonable. Public service levels, economic growth and income and demographics also contribute to other related influences on health cost.

#### 3.4.1. The Level of Public Services

To meet citizens’ direct and specific needs, the relevant departments provide public services for residents under the constraints of relevant laws. Studies have shown that air pollution has a significant impact on health costs. However, within a country, the significance is also different due to the regional differences in the level of public services. With the addition of public service factors, the impact of environmental pollution on national health is reduced to some extent. Therefore, when measuring the impact of air pollution on health expenditure, the level of public services cannot be ignored. Scholars typically choose education, infrastructure, and environmental governance inputs (or the provision of health services). By using the threshold regression method to introduce public service variables, it is concluded that the threshold number of different public services on the impact of environment pollution on health costs is different. Among them, three thresholds are generated by education, and one is generated by urban infrastructure construction and environmental protection. Through the use of the individual fixed effect model, the study concluded that, when economic development, public health, environmental protection, green and other factors are added, the influence degree of air pollution is significantly weakened, and the improvement of economic development and public service level is helpful in reducing the risk of air pollution for health damage (Sun and Li, 2017) [28]. The improvement of local public services can significantly reduce health expenditure (Li et al., 2020) [53].

#### 3.4.2. Economic Growth and Resident Income Level

The interactions between economic growth, environmental pollution, and health costs are multiple and important. The progress of science and technology and economic growth is accompanied by the increase of income and the improvement of the level of medical and health services and medical progress. Scholars in the field of economics mainly focus on two aspects of this interaction.

On the one hand, they focus on the relationship between economic growth and environmental pollution, mainly the relationship between economic growth and air pollutants (Nitrogen oxides, Sulfur oxides, Particulate matter, Industrial waste gas), which is based on the inverted U-shaped hypothesis of the environmental Kuznets curve. GDP per capita has a positive and statistically significant impact on the global panel carbon emissions using data from 58 selected countries (Kais and Sami, 2016) [54]. A bidirectional causal relationship between carbon dioxide emissions and economic growth was established by studying data from a global panel of 51 countries during the period 1995–2013 (Chaabouni et al., 2016 [55]; Sharif, 2012 [56]).

On the other hand, they focus on the study of economic growth and health costs. The impact of economic growth on health care expenditure can be divided into substitution effect and income effect. Substitution effect means that while people work hard to create economic benefits, medical care expenditure increases due to work pressure and other reasons. The income effect means that better medical services can be provided with economic development, and residents will spend more on medical care for their own health. Taking the urban population of 29 Chinese provinces and regions as the research object, it is concluded that the long-term elasticity of health costs to economic growth is 1.66, significantly greater than 1, indicating that the substitution effect of economic growth exceeds the income effect, making the medical care expenditure rise (Yang et al., 2013) [25]. By applying fixed effects, model analysis of environmental pollution and economic growth and medical and health services to the influence of different regional residents of public health and its differences, it is concluded that the eastern and central parts of the public health and economic growth present an inverted u-shaped relationship, the environmental pollution can reduce health costs and health services caused by residents’ health risks (Qu et al., 2018) [57].

#### 3.4.3. Population Structure

Demographic structure raises questions such as the proportion of people over 65, life expectancy, and infant birth and death rates. It is easy to see that as the birth rate declines and the life expectancy of the population increases, per capita health expenditure increases (Apergis et al., 2018) [51]. The elderly population over 65 years of age is more vulnerable to environmental damage, and the corresponding health expenditure is higher (An and Heshmati, 2019) [7]. Using panel data of 20 OECD countries, Hitiris and Posnett (1992) [58] confirmed that population had a positive and statistically significant impact on the impact of air pollution on health costs from 1960 to 1987.

## 4. Discussion

The global literature on air pollution and health costs involves the selection of air pollutants, health estimation models and the application of relevant empirical measurement methods, and puts forward policy recommendations according to the actual situation of each country. However, no matter which research method is chosen, if the emission of polluting gases is not properly controlled and the corresponding standards are not established, the emission of air pollutants will cause huge losses to the economy. Chen et al. (2010) [18] also used the voluntary payment method to study the economic losses caused by near-surface ozone pollution to urban and rural residents in Shanghai in 2008 and concluded that the annual economic losses attributable to health were 32.42 billion yuan (95%CI: 10.80–59.23). Cao and Han (2015) [6] concluded that the total health costs caused by haze increased year by year, from 3.085 billion yuan in 2003 to 11.136 billion yuan in 2013. Chen et al. (2020) [19] studied the health loss caused by PM2.5 concentration in the Beijing–Tianjin–Hebei region in 2017 by using the benefit conversion method, and the economic loss in the three regions was 33.391 billion yuan, 210.9 billion yuan and 16.9 billion yuan, respectively. It is therefore urgent to adopt the necessary policies to reduce air pollution, both for human health and for global economic development.

Although academic studies on the relationship between air pollution and health costs have made some achievements, there are still some shortcomings. Firstly, scholars have not fully considered individual avoidance behaviors. For example, due to the rapid development of the light industry, the availability of dust masks and lampblack masks (such as KN100 can effectively prevent ultrafine dust rate above 99.97%) has greatly increased; the public awareness of epidemic prevention and spontaneous health risk avoidance has also been greatly improved under the influence of COVID-19, so the impact of air pollution on health costs has been alleviated and improved to a certain extent. Secondly, due to the time delay of human body damage caused by air pollution, in the empirical analysis, although some scholars took this time delay into consideration in the selection and design of the econometric model, quite a few scholars did not take it into full consideration, so that the conclusions reached have errors. Finally, there is a certain proportion of health costs caused by personal habits. Therefore, the simple use of the original data obtained in the statistical yearbook as the health costs caused by the air pollution are also a reason for the errors in the empirical results.

## 5. Conclusions 

The essence of the study of the health costs of air pollution is to explore the impact of environmental pollution on the inhabitants of the Earth’s ecosystem and the extent of the impact. Therefore, the author believes that the future research direction of environmental pollution and health costs should improve the deficiencies and make innovations in the selection of proxy variables of environmental pollution. At present, scholars’ choices of proxy variables for environmental pollution are relatively simple, focusing on air pollutants. As science and technology progress, pollution gas monitoring technology has been developed and improved. However, in contrast, the safety of water quality or soil pollution cannot be solved by the individual through effective prevention, and water pollution or soil pollution caused by health costs cannot be ignored. In most cases, especially in rural areas and remote areas, people have no way of knowing whether the water or soil quality is contaminated or contains substances that are harmful to human health. Therefore, the impact of water quality or soil pollution on health costs has become increasingly prominent and will be the focus of future research.

## Figures and Tables

**Table 1:** Classification of air pollution indicators in relevant studies.

Category	Exhaust Gas Index	Air Quality Index	Indirect Indicators
Variable	CO_2_, SO_2_, smoke dust, nitrogen oxides, inhalable particulate matter, etc.	PM10, PM2.5, AQI, etc.	Energy structure, vehicle ownership, mask purchase, etc.
Characteristics	Industrial waste gas contains many types of gases, which makes the analysis more comprehensive	The smaller the particle size is, the more likely it is to absorb more harmful substances; It can be used as a comprehensive index to better reflect air quality	These indicators are indirectly related to air pollution and are also important indicators
Harm	It can cause temporary pathological changes in human respiratory, blood, liver, and other systems and organs	Injury alveolar and mucous membrane, cause bronchial and lung inflammation	Such as car exhaust contains harmful substances lead, human body after inhalation cannot be discharged through the body system

**Table 2:** Studies on the impact of air pollution on health costs based on bivariate and its extended model.

Indicators	Author, Time	Object	The Dependent Variable	The Independent Variables	The Empirical Methods	Main Conclusion
Exhaust gas index	Badulescu et al., 2019 [39]	2000–2014. 28 European Union countries	Per capita expenditure on health	Per capita GDP, per capita CO_2_ emissions, environmental spending, per capita renewable energy consumption	Panel autoregressive distributed lag method	Regarding the impact of carbon dioxide emissions on healthcare expenditure, it is found that there is a negative effect in the short term and a positive effect in the long term
	Cui et al., 2016 [38]	2006–2012. China	Per capita health care consumption expenditure	Waste water, industrial solid emissions, SO_2_ emissions, per capita health insurance premiums	Individual fixed effects model	Air pollution has a positive correlation with per capita health care consumption expenditure and a negative correlation with per capita commercial health insurance premium
	Zaidi and Saidi, 2018 [40]	1990–2015. Sub-Saharan Africa	Combined public and private spending on health	CO_2_ emissions, real GDP per capita	ARDL, VECM	Economic growth has a positive impact on the ecological environment, while CO_2_ emission and NO_2_ have a long-term negative impact on the ecological environment
	Narayan and Narayan, 2008 [9]	1980–1999. 8 OECD countries	Real per capita health expenditure	Real per capita income, nitrogen oxide emissions, sulfur oxide emissions, carbon monoxide emissions	Panel co-integration method	Short-term elasticity shows that income and carbon monoxide emissions have a statistically significant positive effect on health spending
	Yahaya et al. (2016) [41]	1995–2012, 125 developing countries	Per capita actual health expenditure	NO_2_, CO_2_, SO_2_, CO emissions	Panel cointegration test	There is a long-term relationship between per capita health expenditure and all explanatory variables
	Ceylan (2020) [42]	1990–2016, Turkey	Health expenditure	Carbon dioxide, methane, nitrous oxide and fluorinated gas emissions	Support vector regression and multiple linear regression (MLR) models based on Bayesian Optimization	Benefits can be maximized by controlling highly related environmental and health expenditures
	Ouyang H, Zhang Z (2017) [43]	2000–2014, China	Actual per capita medical and health expenditure of urban residents	Environmental quality	Non spatial panel model and spatial panel model	The deterioration of environmental quality will stimulate residents’ demand for medical and health services by affecting residents’ health level, and this impact will increase with the increase of population support burden.
	Alimi et al. (2019) [11]	1995–2014, ECOWAS	Health expenditure	Per capita carbon emissions, per capita income	GMM	At the level of 5% of conventional medical expenditure, the positive value of carbon emission coefficient is significant
	Xu et al., 2019 [37]	2005–2016. China	Per capita expenditure on health	Per capita income, per capita industrial emissions	Bayesian quantile regression	The impact of industrial air pollution on health care expenditure is doubly heterogeneous, and there are significant differences in the awareness of environmental pollution and health problems among residents in high, middle and low income regions.
Air quality index	Mao and Huang, 2016 [44]	2003–2013. China	Health spending	PM10, Sulphur dioxide and soot emissions, and the level of public service supply	Threshold regression model	There is a positive correlation between environmental pollution and health expenditure. The public service variable has a threshold effect on the health expenditure of environmental pollution
	Yang and Zhang, 2018 [45]	2007–2009. China	Family health cost	Environmental pollutant concentration and investment capacity	Ordinary least squares	For every 1% increase in annual exposure to fine particulate matter (PM2.5), household health spending increases by 2.942%
	Li G, He R (2019) [10]	2002–2015, China	Average outpatient visits of residents	PM2. 5. Average mass concentration and PM2 5 maximum mass concentration	Spatial Dobbin model	Lagging phase I PM2 The average mass concentration has a significant impact on the number of visits per capita
	Li and Han, 2015 [46]	2001–2010. China	Expenditure on medical and health care for urban residents	PM2.5, Per capita disposable income, elderly and young dependency ratio	GMM	Smog pollution increases the health costs of urban residents; The impact on the health expenditure of the elderly and the young is more obvious
	An and Heshmati, 2019 [7]	2010–2017. South Korea	Health spending	Air pollutant	Random-effects model	Three air pollutants, NO_2_, O_3_, and PM10, have significant positive effects on health care expenditure, respectively
Indirect indicators	Zhang, 2017 [47]	2013–2014. China	Daily purchase of mask quantity	AQI, Weather, holidays	Multinomial logit model; Poisson model	A 100-point increase in the Air Quality Index (AQI) increased total consumption of masks by 54.5 percent and PM2.5 masks by 70.6 percent
	Shahzad et al., 2020 [48]	1995–2017. Pakistan	Health spending	Carbon emissions, economic growth, information and communication technology and renewable energy consumption	Typical cointegral regression, dynamic OLS, and fully modified OLS	Economic growth and carbon dioxide emissions have a positive impact on health expenditure, while information and communication technology and renewable energy consumption have a negative impact on health expenditure

## Data Availability

Not applicable.
